# Compliance of healthcare workers in a psychiatric inpatient ward to infection control practices during the COVID-19 pandemic: a participant observation study supplemented with a self-reported survey

**DOI:** 10.1186/s12879-024-09429-3

**Published:** 2024-06-17

**Authors:** Leonia Hiu Wan Lau, Queenie Shing Kwan Lam, Minnie Mei Yi Siu, Tiffany Sze Ki Tang, Lorna Kwai Ping Suen, Simon Ching Lam

**Affiliations:** 1 School of Nursing, Tung Wah College, Kowloon, Hong Kong SAR China; 2 School of Nursing, The Hong Kong Polytechnic University, Kowloon, Hong Kong SAR China; 3 School of Nursing, Union Hospital, New Territories, Hong Kong SAR China

**Keywords:** Psychiatric ward, Healthcare workers, Observational study, COVID-19, Standard precautions, Self-reported survey

## Abstract

**Background:**

As an emerging infectious disease with a heterogenous and uncertain transmission pattern, coronavirus disease 2019 (COVID-19) has created a catastrophe in healthcare-associated infections (HAIs) and posed a significant challenge to infection control practices (ICPs) in healthcare settings. While the unique characteristics of psychiatric patients and clinical settings may make the implementation of ICPs difficult, evidence is lacking for compliance with ICPs among healthcare workers (HCWs) in a psychiatric setting during the COVID-19 pandemic.

**Methods:**

A cross-sectional multi-method study based on participant unobtrusive observation coupled with the completion of a self-administered ICP survey was conducted to assess compliance with ICPs among HCWs in a psychiatric inpatient ward in a regional hospital. An online checklist, called eRub, was used to record the performance of HCWs in hand hygiene (HH) and other essential ICPs. Furthermore, a well-validated questionnaire (i.e., Compliance with Standard Precautions Scale, CSPS) was used to collect the participants’ self-reported ICP compliance for later comparison.

**Results:**

A total of 2,670 ICP opportunities were observed from January to April 2020. The overall compliance rate was 42.6%. HCWs exhibited satisfactory compliance to the wearing of mask (91.2%) and the handling of clinical waste (87.5%); suboptimal compliance to the handling of sharp objects (67.7%) and linen (72.7%); and poor compliance to HH (3.3%), use of gloves (40.9%), use of personal protective equipment (20%), and disinfection of used surface/area (0.4%). The compliance rates of the nurses and support staff to HH were significantly different (χ^2^ = 123.25, *p* < 0.001). In the self-reported survey, the overall compliance rate for ICPs was 64.6%.

**Conclusion:**

The compliance of HCWs in a psychiatric inpatient ward to ICPs during the COVID-19 pandemic ranged from poor to suboptimal. This result was alarming. Revisions of current ICP guidelines and policies that specifically target barriers in psychiatric settings will be necessary.

**Supplementary Information:**

The online version contains supplementary material available at 10.1186/s12879-024-09429-3.

## Introduction

Healthcare-associated infections (HAIs), also known as nosocomial infections, are acquired by patients during their stay in hospitals or healthcare facilities. HAIs, particularly infections with pandemic potential, e.g., influenza or severe acute respiratory syndrome (SARS), cause significant morbidity and mortality among infected individuals and increase the economic burden on society [1]. Healthcare workers (HCWs), who are likely to be exposed to a variety of exogenous microorganisms during patient care, act as a major source of HAI transmission. In this regard, infection control practices (ICPs) have long been introduced and implemented to control HAIs. ICPs are designed to disrupt the transmission of microorganisms via contact, respiratory droplets, airborne spread, and common vehicles; they are considered effective and essential approaches for breaking the chain of infection in healthcare settings [2, 3]. By considering all patients as susceptible, ICPs are implemented in all healthcare processes. They consist of two components: standard and transmission-based precautions. Standard precautions are minimum-level infection prevention practices applied to all patients; they include hand hygiene (HH), use of personal protective equipment (PPE), respiratory hygiene, sharps safety, cleansing and disinfection, and waste disposal [4]. Meanwhile, transmission-based precautions are practices used in addition to standard precautions for patients who are known or suspected to be infected or colonized with epidemiologically important or highly transmissible pathogens, e.g., tuberculosis, influenza, and methicillin-resistant *Staphylococcus aureus* [4]. Approximately 10–70% of HAIs can be prevented through the effective implementation of ICPs [5]. 

Coronavirus disease 2019 (COVID-19) is an emerging infectious disease caused by a novel coronavirus called “SARS-CoV-2”. COVID-19 was first identified in Wuhan, China in December 2019 and rapidly became a global threat [6], affecting 770 million people worldwide and causing over 6.9 million deaths as of 2023 [7]. As an emerging infectious disease with a heterogenous and uncertain transmission pattern, COVID-19 created a catastrophe in HAIs and posed a significant challenge to ICPs in healthcare settings. The transmission of SARS-CoV-2 in healthcare settings has been reported in many countries [8, 9]. The COVID-19 pandemic has been associated with an increased rate of HAIs [10]. While the COVID-19 pandemic highlighted the need to strengthen current ICPs, discrepancies exist between recommendations from the international guidelines (i.e., guidelines from the World Health Organization (WHO), the US Centers for Disease Control and Prevention (CDC), and the European Centre for Disease Prevention and Control (ECDC)) and the national guidelines from low- (e.g., India), middle- (e.g., China) and high-income (e.g., Australia and the United Kingdom) countries [11, 12]. Furthermore, gaps always occur between the ICP guidelines and their implementation. Suboptimal compliance with ICPs among HCWs is always one of the concerns. Noncompliance to ICPs is common in hospitals and healthcare facilities, such as nursing homes [13, 14], in which the compliance rate varied among different occupational groups and ICP components [15]. Worldwide, the compliance rate with ICPs is less than 50% in high-income and low-income settings [16–18]. In Hong Kong, the overall compliance rate to ICPs is around 57.4–58.9% (a compliance rate ≥ 90% indicates an optimal level) [19, 20]. 

Although an increasing number of studies have examined compliance with ICPs among HCWs, most of these studies have focused on non-psychiatric ward settings, such as intensive care units, accident and emergency departments, or surgical wards. No studies have yet investigated compliance with ICPs among HCWs in psychiatric settings. ICP guidelines in psychiatric settings are generally the same as those in general ward settings. However, the unique characteristics of psychiatric patients and ward settings may make implementation difficult [21, 22]. Attention in psychiatric care may shift to the safety maintenance and management of psychiatric problems. Different unpredictable issues (e.g., violence, emotional outbursts, and self-harm behavior) may frequently exist in psychiatric wards, and they can hinder the proper implementation of ICPs. Some infection control equipment, such as alcohol hand rub at each bedside or built-in sink, may be limited because of concerns about ingestion of alcohol by patients with a history of substance abuse [21]. Moreover, psychiatric patients generally have a higher incidence of chronic infections related to substance abuse and unprotected sexual behavior, such as human immunodeficiency virus (HIV) infection, hepatitis B and C, and tuberculosis, making them more susceptible to HAIs [22]. Under such background, our study aimed to investigate compliance with ICPs among HCWs in a psychiatric setting during the COVID-19 pandemic.

## Methods

### Study design and settings

A cross-sectional multi-method study based on participant unobtrusive observation coupled with the completion of a self-administered survey was conducted to assess compliance with ICPs among HCWs in a psychiatric setting. We followed the guideline of STrengthening the Reporting of OBservational studies in Epidemiology (STROBE) for reporting. This study was conducted in a psychiatric inpatient acute admission ward in a hospital in Hong Kong during the first local wave of the COVID-19 pandemic between January and April 2020. The study ward had 48 hospital beds and around 31 HCWs working in it. All the patients in the study ward were admitted with acute mental disorders (e.g., schizophrenia, bipolar disorder, dementia, and personality disorder). Some of them also had medical comorbidities (e.g., diabetes mellitus, hypertension, and arthritis). The ward had four cubicles in, each of which accommodated ten patients. The patients in Cubicles 1 and 4 were generally ambulatory and could perform activities of daily living by themselves. Cubicle 2 consisted of patients aged under 18 years and over 65 years who were physically weak and needed more medical attention (e.g., history of epilepsy, history of falls, and oxygen therapy required). Cubicle 3 comprised newly admitted patients who needed cohort nursing and close observation. In addition, three side rooms (called Cubicle 5 in this study) were reserved for accommodating patients with four-limb restraints, high violence risk, or isolation needs (e.g., fever). These arrangements were changed (except for Cubicle 5) in February 2020 for surveillance purposes during the COVID-19 pandemic. All newly admitted patients were arranged to stay in Cubicles 1 to 3 in accordance with the recommended surveillance period. Patients who had completed surveillance were transferred to Cubicle 4. The use of Cubicle 5 remained unchanged, to which newly admitted patients with travel history and/or abnormal X-ray reports (suspected COVID‐19 cases) were allocated.

### Sample and sampling size

Using convenience sampling, all the registered nurses, enrolled nurses, and clinical support staff, except for the administrative nursing staff working in the study ward, were recruited. In accordance with the World Health Organization (WHO) guidelines on the minimal number of observations required to determine HH compliance [23], 200 opportunities were observed in each unit (interpreted as cubicle in this current study), resulting in about 1,000 opportunities observed in the study ward. The observation schedule was constructed on the basis of the observer’s duty roster. A pilot study indicated that about 18 opportunities were observed in 3 observation sessions (20 min per session). Thus, at least 12–14 weeks (observed before and after each shift) were required to complete the sufficient observations. Following the 14-week observation period, the observed staff members were invited to respond to a questionnaire on ICP compliance. This approach allowed overall comparison between the self-reported retrospective (i.e., recalling their previous ICPs) and observational data.

### Observational measurement of ICPs

In the unobtrusive participant observation, ICPs among HCWs were observed by high-resolution closed-circuit television. Observation was started at any opportunity that ICPs should be performed. As such, no prior notification was given for each observation. The observer (one of the researchers) was a registered psychiatric nurse who was trained and familiar with the ICP concept, and the setting and routines of the psychiatric ward being studied. The ICP episodes of the participants were recorded using a handheld electronic device installed with a software platform called eRub (http://www.flowmedik.com, SAG Flowmedik Oy, Finland). eRub is an observation checklist that comprises two sections: compliance with HH and other ICP items, which were constructed on the basis of WHO’s “Five Moments for HH” [23] and the infection control guidelines laid down by the Centers for Disease Control and Prevention (CDC) [24]. HH activities, including timing (i.e., before or after patient contact, before aseptic task, after body fluid exposure risk, and after contact with patient surroundings) and duration (i.e., either washing hands > 20 s with water and soap or rubbing hands > 20 s with alcohol-based hand rub), were observed and comprehensively recorded [23, 24]. Meanwhile, other ICP items (i.e., respiratory hygiene; disinfection of used surfaces/equipment; use of PPE; and handling of linen, clinical waste, and sharp objects) were observed and rated when opportunities occurred [4]. HH performance was rated using a three-point scale [0 = missed performing, 1 = performed but inadequate (< 20 s), and 2 = well performed (> 20 s)]. The performance of other ICPs was rated using a three-point scale, i.e., 0 = missed performing (did not perform the described ICP), 1 = improperly performed (performed the described ICP with less than 80% correctness), and 2 = properly performed (performed the described ICP with over 80% correctness) (refer to supplementary information for the checklist) [14]. The content validity indices based on six infection control experts regarding the relevance and adequacy of the items of eRub were greater than 0.83 (range = 0.83–1.00), indicating satisfactory content validity.

### Self-reported Compliance with Standard Precautions Scale (CSPS)

CSPS was employed to capture self-reported compliance with ICPs [25]. CSPS comprises 20 items, covering 5 dimensions/areas of ICPs (i.e., use of protective devices, disposal of sharp instruments and biological waste, decontamination of spills and used equipment, and prevention of cross-infection). Each item was rated using a 4-point adjectival scale (i.e., never, seldom, sometimes, and always). The total score of the 20-item CSPS ranged from 0 to 20, with a higher score indicating better compliance. The scoring, compliance rate calculation, and recoding method of CSPS have been described elsewhere [25, 26]. The overall compliance rate can be calculated by averaging the compliance rate of all the 20 items, and it can be described as “optimal” (> 90%), “satisfactory” (80–89%), “suboptimal” (50–79%), and “poor” (< 49%). The psychometric properties of CSPS are satisfactory [25]. CSPS has been validated in Hong Kong and adapted (translated and re-validated) in more than 12 countries [27–31]. 

### Data analysis

Data were analyzed using SPSS®-PC version 26. The compliance with HH and other ICPs of the participants were summarized using descriptive statistics. Differences in ICP compliance between nursing and clinical support staff were inferred using chi-squared and Mann–Whitney U tests with statistical significance defined by *p* < 0.05.

### Ethical consideration

Ethical approval was obtained from the Research Ethics Committee of the Hospital Authority, the Human Subjects Ethics Application Review System of the Hong Kong Polytechnic University, and the chief of service and manager of the Department of Psychiatry. Information sheets regarding the nature and period of the study were provided to all the participants in a previous internal conference meeting. Informed consent was obtained from the participants and the respective ward manager before data collection. However, the time when they would be observed was not disclosed. The researchers emphasized that no personal identity was recorded.

## Results

### Result of participant unobtrusive observation

In this study, 2,670 ICP opportunities were observed on the basis of the practices of 21 nurses and 10 clinical support staff during a 14-week period from January 2020 to April 2020. The ICP elements observed included HH [*n* = 1000]; use of gloves [*n* = 296], masks [*n* = 1000], and PPE [*n* = 35]; disinfection of used surface/equipment [*n* = 258]; and handling of linen [*n* = 11], clinical wastes [*n* = 39], and sharp objects [*n* = 31] (Supplementary Table 1). Overall ICP compliance (i.e., proportion of the total observed ICP opportunities for which properly-performed ICP activities were taken) was inadequate (42.6%). In particular, the average compliance rate was 3.3%, 40.9%, 91.2%, 20.0%, 0.4%, 72.7%, 87.5%, and 67.7% for HH, use of gloves, use of a mask, use of PPE, disinfection of used surfaces/equipment, handling of linen, handling of clinical wastes, and handling of sharp objects, respectively (Tables 1 and 2). Significant differences were found in ICP compliance between the nursing staff and the clinical support staff, in which the nursing staff complied to ICPs better than the clinical support staff in all aspects.

**Fig. 1:**
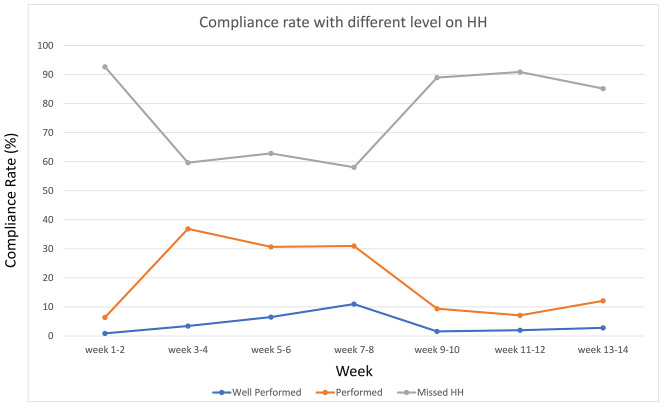
Performance of hand hygiene (HH) practice among healthcare workers

**Table 1:** Performance of hand hygiene (HH) practices among healthcare workers, five moments of indication, and different units (*N* = 1000)

Variables	Total (N = 1000)	Well performed (n = 33)	Performed but inadequate (n = 129)	Missing HH (n = 836)	Statistical test, *p-value*
Profession of healthcare worker:					
Nursing staff, n (%)	238 (23.8%)	27 (11.3%)	63 (26.5%)	148 (62.2%)	χ^2^ = 123.25, *p* < 0.001
Clinical supporting staff, n (%)	762 (69.6%)	6 (0.8%)	66 (8.7%)	690 (90.6%)	
Five moments of HH:					
Before patient contact, n (%)	471 (47.2%)	0	5 (1.1%)	466 (98.9%)	NA
After patient contact, n (%)	405 (40.6%)	19 (4.7%)	73 (18%)	313 (77.3%)	
Before aseptic task, n (%)	0	0	0	0	
After body fluid exposures risk, n (%)	87 (8.7%)	9 (10.3%)	41 (47.1%)	37 (42.5%)	
Patient surrounding, n (%)	35 (3.5%)	5 (14.3%)	10 (28.6%)	20 (57.1%)	
Units:					
Cubicle 1	350 (35.0%)	4 (1.1%)	46 (13.1%)	300 (85.7%)	NA
Cubicle 2	241 (24.1%)	14 (5.8%)	28 (11.6%)	199 (82.6%)	
Cubicle 3	232 (23.2%)	5 (2.2%)	23 (9.9%)	204 (87.9%)	
Cubicle 4	73 (7.3%)	3 (4.1%)	7 (9.6%)	63 (86.3%)	
Cubicle 5 (3 side-room)	104 (10.4%)	7 (6.7%)	25 (24%)	72 (69.2%)	

**Table 2:** Performance of use of mask and gloves and other standard precautions practices among healthcare workers

**Dependent variables**	Total (N = 1000)	**Well Performed**	Performed	Improperly Performed	Missed to performed
**Use of Mask**:	1000	912 (91.2%)	45 (4.5%)	38 (3.8%)	5 (0.5%)
Nursing Staff, n (%)	238 (23.8%)	232 (97.5%)	2 (0.8%)	2 (0.8%)	2 (0.8%)
Clinical supporting staff, n (%)	762 (76.2%)	680 (89.2%)	43 (5.6%)	36 (4.7%)	3 (0.4%)
**Use of Gloves**:	296	121 (40.9%)	18 (6.1%)	102 (34.5%)	55 (18.6%)
Nursing Staff, n (%)	90 (23.8%)	54 (60%)	4 (4.4%)	19 (21.1%)	13 (14.4%)
Clinical supporting staff, n (%)	206 (76.2%)	67 (32.5%)	14 (6.8%)	83 (40.3%)	42 (20.4%)
**Disinfecting used surface**:	258	1 (0.4%)	4 (1.6%)	10 (3.9%)	243 (94.2%)
Nursing Staff, n (%)	6 (23.8%)	1 (16.7%)	1 (16.7%)	0	4 (66.7%)
Clinical supporting staff, n (%)	252 (76.2%)	0	3 (1.2%)	10 (4%)	239 (94.8%)
**Handling of Linen**:	11	8 (72.7%)	1 (9.1%)	2 (18.2%)	0
Nursing Staff, n (%)	1 (23.8%)	1 (100%)	0	0	0
Clinical supporting staff, n (%)	10 (76.2%)	7 (70%)	1 (10%)	2 (20%)	0
**Handling of Clinical Waste**:	40	35 (87.5%)	2 (5.0%)	0	2 (5%)
Nursing Staff, n (%)	34 (23.8%)	32 (94.1%)	1 (2.9%)	0	0
Clinical supporting staff, n (%)	6 (76.2%)	3 (50%)	1(16.7%)	0	2 (33.3%)
**Handling of Sharp**:	31	21 (67.7%)	4 (12.9%)	6 (19.4%)	0
Nursing Staff, n (%)	31 (23.8%)	21 (67.7%)	4 (12.9%)	6 (19.4%)	0
Clinical supporting staff, n (%)	0 (76.2%)	0	0	0	0
**Use of PPE**:	35	7 (20%)	20 (57.1%)	5 (14.3%)	3 (8.6%)
Nursing Staff, n (%)	5 (23.8%)	0	4 (80%)	0	1 (20%)
Clinical supporting staff, n (%)	30 (76.2%)	7 (23.3%)	16 (53.3%)	5 (16.7%)	2 (6.7%)

The level of HH compliance changed with the pandemic intensity level of COVID-19. The HH missing rate was around 90% at Weeks 1 and 2 (before the onset of the 1st wave of outbreak), and dropped to about 60% at Weeks 3–8 (the 1st wave of outbreak started with a sudden increase in the number of confirmed cases) and returned to 90% at Weeks 9–14 (the 1st wave of outbreak was controlled). Inadequately performed and well-performed HH rates were observed with a similar but reversed pattern, rising to the highest in February (Weeks 3–8) and dropping in April (Weeks 11–14) (Fig. 1, Supplementary Fig. 1). While the HH missing rate dropped to about 60% at Weeks 3–8, HH still tended to be inadequately performed (10% for well-performed HH versus 30% for inadequately performed HH). The observed HH opportunities were also recorded on the basis of the definition of the five moments (Table 1). The moments of indication for HH were frequent in “before and after patient contact” (87.8%, *n* = 876). However, the well-performed HH rates for the moment “before patient contact” and “after patient contact” were 0% and 4.7%, respectively. Moreover, 98.9% of the staff missed performing any HH before patient contact. The most compliant moment was “after touching a patient surrounding,” in which 14.3% of the staff performed HH with adequate time (i.e., well-performed HH). During the surveillance period of COVID-19, the observation episodes in some cubicles did not reach 200 because the cubicles sometimes had no patient in there. Differences in HH compliance among HCWs were found in different cubicles. HCWs performed better and missed less HH (missing HH = 69.2%) in Cubicle 5 than in the other cubicles (missing HH = 82.6–87.9%, Table 1). With regard to difference in HH compliance between nursing and clinical support staff, the rates of well-performed HH was 11.3% for the nurses and 0.8% for the support staff (Table 1). Meanwhile, in terms of the rates of well-performed mask and glove usage, the compliance level for the other ICPs also demonstrated a similar pattern as that of HH across the observation period.

### Compliance with ICPs determined using the self-reported survey

A total of 25 self-reported questionnaires (i.e., 10 and 15 from clinical support and nursing staff, respectively) were completed and returned. The response rate was 80.6%. Maternity and long sick leaves were the reasons for the inability to return the questionnaire. The overall compliance rate of HCWs measured via CSPS was 64.6%, in which compliance with HH, face mask use, and glove use were 50.0%, 69.3%, and 76.0%, respectively (Supplementary Table 2). The compliance rates of HH (50.0% in the self-reported survey versus 3.3% in the participant observation study) and glove use (76.0% versus 40.9%) obtained in the self-reported survey were higher than those determined in the participant observation method. By contrast, the compliance rate of face mask use (69.3% versus 91.2%) reported in the survey was lower than that determined in the observation study.

## Discussion

To our knowledge, this study was the first to assess compliance with ICPs among HCWs in a negligent clinical setting (i.e., a psychiatric inpatient ward), by using a combination of participant unobtrusive observation and self-administered survey. The overall ICP compliance was suboptimal (42.6% from the observation method, 64.6% from the self-reported survey). These findings were consistent with previous studies conducted during the COVID-19 pandemic [32–34]. As compared with the ICP compliance during the SARS epidemic in 2003 and the H1N1 influenza (swine flu) pandemic in 2009, the ICP compliance during COVID-19 was decreasing. (35–36) The findings from the observation method suggested that the level of compliance varied among different ICP elements, with the lowest level observed in HH and the highest level observed in face mask use. The level of compliance also varied with the pandemic intensity level of COVID-19, rising to the highest level in late January to February (Weeks 3–8) and dropping in late March to early April (Weeks 11–14). Significant differences in ICP compliance were found between the nursing staff and the clinical support staff.

HH compliance among HCWs in a psychiatric setting was particularly low, with only 3.3% of HCWs demonstrating well-performed HH. A previous local study, which was based on 1,037 observations in 4 clinical areas in an acute hospital (medical wards, surgical wards, accident and emergency department, and intensive care unit) and 2 (medical and surgical wards) in 2 rehabilitation hospitals, showed that overall HH compliance in HCWs was around 74.7% 37. In a recent local study, the HH compliance of HCWs in pediatric units reached 79.8–100% for over 380 observations [38]. The huge difference in ICP compliance between the settings suggested that current ICP guidelines designed for general medical settings might not be directly applicable to psychiatric settings. Their implementation could be potentially challenged by the unique characteristics of psychiatric patients and clinical settings. Our study results also found that “before patient contact” was the moment (indicator) with the poorest HH compliance. This finding was in line with that of Eckmanns et al. [39] Self-protection (instead of patient protection) as the major motivation of HH and the perception that HH is a less effective way to prevent cross-infection could be the reasons behind the results [40]. The high missing HH rate in Cubicles 1, 2, and 3, which had high admission rates during the surveillance period of COVID-19, suggested that the working environment could be another reason that influenced compliance with HH. A study explained HH beliefs in relation to the working environment and reported that nurses are less motivated to perform HH properly because they are too busy and/or dealing with emergencies [41]. By contrast, a relatively low missing HH rate was found in Cubicle 5 (i.e., cases with fever and respiratory symptoms). One possible reason could be that HCWs were highly alert to protect themselves from the high-risk patients in Cubicle 5. This finding further supported that HH is primarily motivated by self-protection, which is plausible and congruent with several previous studies [41–43]. 

Appropriate glove use was only demonstrated in less than half of the observed opportunities (40.9%). In addition, 34.5% of the participants did not change gloves between patients and wore gloves before contacting patients when they were not supposed to contact blood, body fluid, and excreta. HCWs also removed their gloves without performing HH. These observations were consistent with those of Fuller et al. [44]. , in which the participants wore gloves when it was not indicated and their HH compliance rate was significantly lower. The study results suggested that some HCWs might have the misconception that the use of gloves could be a substitute for HH [44]. Similarly, Flores and Pevalin found that participants tended to overuse gloves, which might be associated with the false belief that using gloves could obviate HH [45]. Gloves should never be used to replace effective HH, because they cannot completely prevent the contamination of the hands [46]. Meanwhile, overall compliance to properly-performed mask-wearing was high (91.2%), which could be explained by the changes in perception toward mask-wearing after the SARS epidemic in 2003.

Compliance with HH and the use of masks and gloves appeared to change with COVID-19 pandemic intensity, which rose to the highest level in late January to February (Weeks 3–8, during which the 1st wave of outbreak started with a sudden increase in the number of confirmed cases) and dropped in late March to early April (Weeks 11–14, during which the 1st wave of outbreak was controlled) [47]. Such findings suggested that HCWs adjusted their compliance with ICPs on the basis of their risk perception regardless of the presence of the pandemic [42]. Compared with HH, HCWs generally performed better in all the other ICPs. This result was in line with the current self-reported survey, in which higher compliance rates were obtained with the other ICPs (data not shown). The overall compliance rate measured by CSPS was suboptimal (64.6%) and comparable with those of previous studies. (27, 48–49) The rates of compliance with HH (50%) and glove use (76%) obtained in the self-reported survey were higher than those obtained in the participant observation method. Such discrepancies suggested that HCWs might report the idealized practice that they are expected to perform but not their actual behavior [15]. However, compared with the participant observation method, lower compliance rate for face mask use was found in the current survey (69.3%), particularly in Item 15 (reuse of the surgical mask). The shortage of mask and other PPE during the COVID-19 pandemic may be the reason for the reduced compliance to “not to reuse disposable mask”[53]. Meanwhile, the gaps in knowledge and practices in infection control among HCWs can be another possible reason.

Our study was not without limitations. Although we adopted WHO’s suggestion to observe 200 opportunities, the episodes of other ICPs, such as handling of linen, sharp objects, and clinical waste, and the use of PPE, were few (11–39 opportunities). The results might not be adequate to reflect true compliance with these ICPs among HCWs. Meanwhile, this study employed a quantitative design, and a qualitative inquiry regarding the reasons behind ICP noncompliance among participants (e.g., poor compliance to HH before patient contact and improper use of gloves) was lacking. A future qualitative study may be helpful in exploring barriers that hinder compliance with ICPs in psychiatric settings. Last, psychotherapy and counselling procedures, conducted by healthcare professionals, are frequently utilised in psychiatric inpatient ward. In the assessment of compliance with standard precautions through a self-reported survey, healthcare staff provided responses based on their everyday interactions, without emphasising the implementation of psychotherapy and counselling intervention. Therefore, the effect of these therapies on ICP was uncertain. Despite that, our study provided evidence regarding compliance with ICPs among HCWs in a negligent clinical setting. Compliance with ICPs depends not only on knowledge and skill of ICPs, but more importantly, on complex factors that support implementation, which may be setting-specific (e.g., risk perception, working environment, and motivation of self-protection) [50]. A revision of current ICP guidelines and policies that specifically targets barriers in psychiatric settings will be necessary. Further strengthening existing infection control training programs will be important to address possible knowledge and practice gaps among HCWs. Moreover, our study demonstrated the use of closed-circuit television and the eRub observation checklist to observe ICP compliance, suggesting the potential of incorporating these techniques into current auditing practices to improve ICP compliance. While direct observation is considered the gold standard method for monitoring ICP compliance, a participant unobtrusive observation method through closed-circuit television can minimize the awareness of HCWs when being observed, neutralize the Hawthorne effect of direct observation, and provide a more realistic picture of actual compliance [51]. The use of the eRub observation checklist allows the observer to gather data effectively and saves time for data entry, and thus, feedback can be provided immediately after the audit [52]. 

## Conclusion

Overall compliance with ICPs among HCWs in a psychiatric setting was suboptimal. This finding was alarming, particularly during the COVID-19 pandemic. Compliance with ICPs changed with outbreak intensity. HH compliance was particularly low. Among the five moments for HH, the poorest compliance was observed “before patient contact.” Together with the overuse/misuse of gloves, ICPs among HCWs appeared to be motivated by self-protection rather than patient protection. Moreover, the implementation of ICPs can be potentially challenged by the unique characteristics of psychiatric patients and clinical settings. Revisions of current ICP guidelines and policies that specifically target the barriers in psychiatric settings will be necessary.

## Electronic supplementary material

Below is the link to the electronic supplementary material.

Supplementary Material 1

## Data Availability

The data and materials are available from the corresponding author upon reasonable request.
