# Supplementary material for: Compliance of healthcare workers in a psychiatric inpatient ward to infection control practices during the COVID-19 pandemic: a participant observation study supplemented with a self-reported survey
Source: BMC Infect Dis. 2024 Jun 17;24:592. doi: 10.1186/s12879-024-09429-3 (PMC11181547; doi:10.1186/s12879-024-09429-3)
Supplement: Supplementary file 1 — Supplementary Material 1 [file 12879_2024_9429_MOESM1_ESM.docx]

**Supplementary information of the checklist**

**Infection control practice observational checklist powered by eRub** (digital service provided by SAG Flowmedik Oy, <https://www.flowmedik.com/en/>)

**Section 1: Demographics**

Uni being observed: ___________

Profession*: Allied health professionals / Doctors / Nurses / Health workers / Personal Care workers

**Section 2: Five moments of hand hygiene**

| **Items** | **Hand disinfectant usage time is recorded (in seconds)^** |
| --- | --- |
| 1. Hand hygiene performed before patient contact |  |
| 2. Hand hygiene performed after patient contact |  |
| 3. Hand hygiene performed before aseptic task ... |  |
| 4. Hand hygiene performed after body fluid exposure risk |  |
| 5. Hand hygiene performed after contact with patient surroundings |  |
| 6. Hand disinfectant dispenser near: Yes / No | |

Remarks: ^0 second means missing the hand hygiene practice

**Section 3: Other Practice of Standard Precautions#**

| **Items** | **Not Done** | **Improperly done** | **Properly done** | **Not applicable** |
| --- | --- | --- | --- | --- |
| 7. Respiratory hygiene | 0 | 1 | 2 | NA |
| 8. Handling sharp equipment | 0 | 1 | 2 | NA |
| 9. Decontaminating equipment | 0 | 1 | 2 | NA |
| 10. Waste management | 0 | 1 | 2 | NA |
| 11. Personal protective equipment | 0 | 1 | 2 | NA |
| 12. Environmental cleanliness | 0 | 1 | 2 | NA |

13. Add risk factor observation:

🞏 Ring / 🞏 Wristband or watch / 🞏 Artificial nails / 🞏 Long nails / 🞏 Nail polish

🞏 Other ____________________

14. Add observation of gloves:

🞏 No gloves used / 🞏 Gloves used correctly / 🞏 Gloves used incorrectly

Remarks:

*Professional: Allied Health professionals = Physiotherapist, Occupational Therapist, Radiographer, Speech therapist, and other appropriate.

Doctors: Licensed medical practitioners including traditional Chinese Medicine practitioners, and medical students.

Nurses: Licensed nursing professionals and nursing students.

Health workers: Assistants with certificates or in training, e.g., Health Care Assistants, Physiotherapists Assistants or Occupational therapists Assistants or Radiographers Assistants.

Personal Care Workers: Provision of direct nursing and patient care with training (but without a certificate), tasks mainly on client’s personal and household hygiene, meal preparation and etc.

#Not Done (0) = missed performing (did not perform the described infection control practice)

Improperly done (1) = improperly performed (incorrectly performed the described infection control practice)

Properly done (2) = performed (performed the described ICP with over 80% correctness)

Not applicable (3) = not applicable for the observed situation

……………………………………………………………………………………………………

Author retains the copyright of the above eRub checklist, and reproduction of CSPS is available with Author’s permission only (**Lam, S. C.**).

Source of eRub checklist: Au, J. K. L., Suen, L. K. P., & **Lam, S. C.** (2021). Observational study of compliance with infection control practices among healthcare workers in subsidized and private residential care homes. *BMC Infectious Diseases, 21*, 1-11. <https://doi.org/10.1186/s12879-021-05767-8>

**Supplementary Table 1. Number of infection control practice (ICP) opportunities observed**

| ICP items | Total | Nursing Staff  n (%) | Clinical Supporting Staff  n (%) |
| --- | --- | --- | --- |
| Hand hygiene | 1000 | 238 (24.0%) | 762 (76.0%) |
| Use of gloves | 296 | 90 (30.4%) | 206 (69.6%) |
| Use of face mask | 1000 | 238 (24.0%) | 762 (76.0%) |
| Disinfecting used surfaces/equipment | 258 | 6 (2.3%) | 252 (97.7%) |
| Handling of linen | 11 | 1 (9.0%) | 10 (91.0%) |
| Handling of clinical waste | 39 | 33 (84.6%) | 6 (15.4%) |
| Handling of sharps | 31 | 31 (100%) | 0 |
| Use of personal protective equipment | 35 | 5 (14.3%) | 30 (85.7%) |

**Supplementary Table 2. Self-reported compliance rate of infection control practices (ICPs) among healthcare workers based on Standard Precautions Scale (N=25)**

| **Variables** | **Overall (N=25)** | **Nursing Staff (N=15)** | **Clinical supporting staff (N=10)** | **Statistical test, *p-value*** |
| --- | --- | --- | --- | --- |
| Total compliance rate | 64.6% | 54.3% | 80.0% | *U*=22.0, *p*<0.003 |
| Compliance rate on HH | 50.0% | 38.3% | 67.5% | *U*=30.5, *p*<0.010 |
| Compliance rate on the use of mask | 69.3% | 62.2% | 80.0% | *U*=44.0, *p*<0.059 |
| Compliance rate on the use of gloves | 76.0% | 60% | 100% | *U*=15.0, *p*<0.001 |

**Supplementary Figure 1. Compliance rate of properly performed infection control practice (ICP) among healthcare workers**


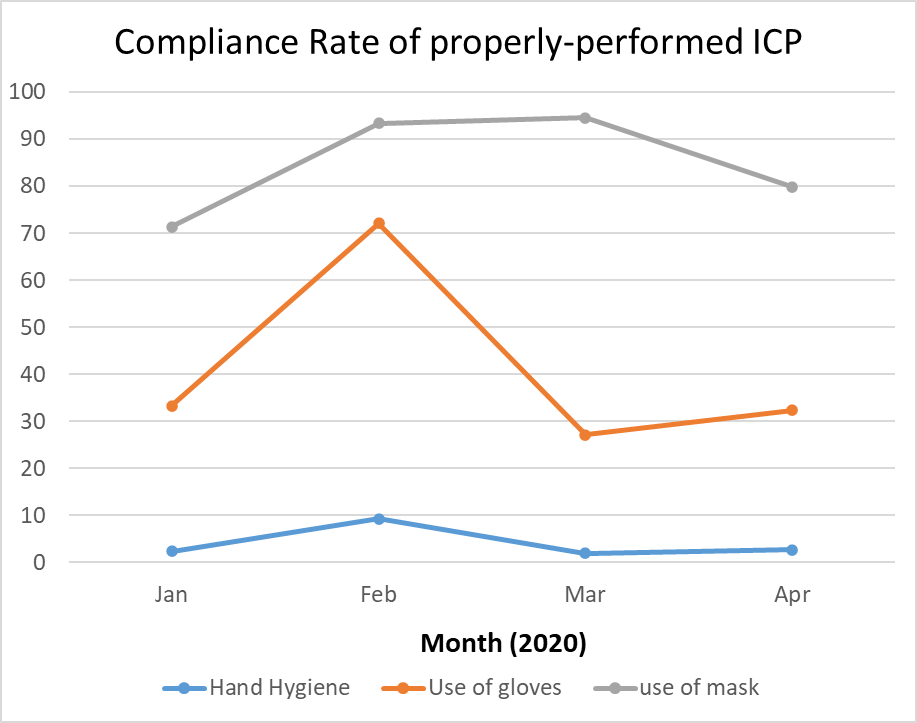


**Compliance Rate (%)**
